# Association Between Psychosocial Characteristics and eHealth Literacy: Cross-Sectional Study of Hybrid Secondary Prevention in Mental Health

**DOI:** 10.2196/73697

**Published:** 2025-10-20

**Authors:** Johannes Stephan, Jan Gehrmann, Ananda Stullich, Monika Sinha, Matthias Richter

**Affiliations:** 1 Chair of Social Determinants of Health, TUM School of Medicine and Health, Technical University of Munich, Am Olympiacampus 11, Munich, 80809, Germany, 0049 89 289 24191; 2 Institute of General Practice and Health Services Research, Department Clinical Medicine, TUM School of Medicine and Health, Technical University of Munich, Munich, Germany; 3 Department of Prevention and Rehabilitation, German National Pension Fund, Berlin, Germany

**Keywords:** eHealth literacy, psychosocial, hybrid prevention, mental health, digital health

## Abstract

**Background:**

Increasing psychosocial burdens, such as stress and anxiety, underscore the need for accessible and effective prevention programs. Hybrid approaches, combining in-person and digital components, aim to reduce barriers and enhance flexibility. However, their effectiveness depends on participants’ eHealth literacy, which is associated with their ability to engage with digital tools. Understanding how psychosocial characteristics relate to eHealth literacy can provide insights for improving intervention design.

**Objective:**

This study uses cluster analysis to explore the relationship between psychosocial characteristics and eHealth literacy in a hybrid mental health prevention program. By identifying distinct psychosocial profiles and analyzing their differences in eHealth literacy levels and patterns, this person-centered approach enables a nuanced understanding of eHealth literacy disparities beyond traditional variable-centered linear models. In addition, the study examines how sociodemographic variables are associated with eHealth literacy, providing insights into the role of psychosocial diversity in hybrid prevention programs.

**Methods:**

A cross-sectional study was conducted with participants of the RV Fit Mental Health intervention (January 2024–December 2024). Psychosocial characteristics, including anxiety, depression, optimism, pessimism, quality of life, self-efficacy, stress, and work ability, were assessed alongside eHealth literacy (eHealth Literacy and Use Scale [eHLUS], eHealth Literacy Scale [eHEALS]). To identify distinct psychosocial profiles, cluster analysis was used. A generalized linear model was applied to analyze associations between cluster membership, eHealth literacy, and sociodemographic variables. Finally, correlation matrices were used to further explore the relationships between psychosocial characteristics and eHealth literacy.

**Results:**

A total of 173 participants were included. Four clusters were identified based on psychosocial characteristics. Significant associations were found between cluster membership and eHealth literacy, including the overall eHLUS score (*P*=.004) and its dimension of autonomous use and technical access (*P*=.003). Cluster 3 (n=65) had the most favorable psychosocial characteristics and the highest eHealth literacy levels. Cluster 4 (n=36) exhibited the least favorable psychosocial characteristics but mid-range eHealth literacy levels. Cluster 2 (n=45) showed the lowest eHealth literacy levels despite a mid-range psychosocial profile. Cluster 1 (n=27) demonstrated mid-range eHealth literacy levels and mid-range psychosocial characteristics. In addition, age and subjective socioeconomic status were significantly associated with eHealth literacy levels. Beyond the identified clusters, significant correlations were observed between individual psychosocial characteristic variables and eHealth literacy.

**Conclusions:**

The cluster analysis identified distinct psychosocial profiles with varying levels of eHealth literacy, demonstrating that psychosocial characteristics are associated with eHealth literacy in diverse ways. These findings underscore the need to consider subgroup-specific needs in hybrid prevention programs. Certain groups may require additional support to effectively navigate eHealth tools. These findings emphasize the relevance of tailored intervention strategies that account for psychosocial diversity in eHealth engagement.

## Introduction

The increasing prevalence of mental health issues, such as chronic stress, depression, and anxiety disorders, poses a significant challenge to the health care system [1-6], particularly in designing effective prevention programs. Early interventions in the form of prevention programs have demonstrated long-term benefits for participants’ well-being while simultaneously reducing health care costs [7-12].

The digitization of prevention programs offers new opportunities to enhance the efficiency and accessibility of these programs [13]. Digital and hybrid prevention approaches provide innovative solutions by offering greater adaptability and promoting mental health. Hybrid prevention programs combine in-person and digital components, reducing location- and time-related barriers while retaining the benefits of face-to-face interactions. A key advantage of hybrid interventions is the involvement of health care professionals, who can provide guidance and support in navigating digital components. This flexibility enhances their effectiveness by catering to individual participants’ needs [14,15].

The success of hybrid prevention programs depends on their structure, content, and the participant’s ability to engage effectively with the digital components [16,17]. This ability, called eHealth literacy, encompasses the skills required to locate, understand, and apply digital health information [18]. A lack of these skills can limit active participation and reduce the effectiveness of interventions, highlighting the critical role of eHealth literacy in hybrid prevention programs [19,20]. eHealth literacy is widely recognized as crucial for engaging with digital health services [21,22].

To better understand this ability, it is necessary to examine not only whether eHealth literacy is present, but also how it is structured and shaped by contextual and individual factors. The Transactional Model of eHealth Literacy (TMeHL) conceptualizes eHealth literacy as a dynamic and multifaceted capability that extends beyond information retrieval. It emphasizes eHealth literacy as the result of interactions between individual abilities, contextual conditions, and task demands, thereby capturing the evolving and reciprocal character of digital health engagement [23].

Despite this conceptual complexity, research has increasingly examined ways to improve eHealth literacy, including a growing number of targeted interventions [24,25] and has found it to be associated with the acceptance and use of digital health tools [18,26]. Moreover, existing studies indicate that social determinants, such as age, gender, educational level, migration background, and socioeconomic status, are significantly associated with eHealth literacy, highlighting their importance in implementing tailored, needs-based interventions [27-29]. Given that social determinants are linked to differences in individuals’ ability to navigate digital health resources, it is important to consider that psychosocial characteristics, including burdens such as anxiety, depressive symptoms, and stress, as well as resources such as self-efficacy, may be associated with motivation and with varying levels of eHealth literacy. To clarify this conceptual distinction, psychosocial characteristics refer to intraindividual psychological traits and states [30], whereas social determinants of health describe the broader societal conditions in which people are born, grow, live, work, and age, and which affect health outcomes and access to resources [31]

It is important to note that social determinants of health such as subjective socioeconomic status or employment status may be associated with psychosocial characteristics; for instance, by contributing to stress, anxiety, or reduced self-efficacy in situations of financial strain or job insecurity [32]. While the association between social determinants and eHealth literacy is well documented [33], the specific role of psychosocial characteristics remains underexplored. Given that emotional burden and motivational resources may be related to individuals’ ability and readiness to access, process, and use digital health information, this perspective offers a complementary and necessary extension of existing research. This raises critical questions about whether individuals with unfavorable psychosocial characteristics exhibit lower levels of eHealth literacy and how such variations impact their engagement with hybrid prevention programs. While variable-centered approaches, such as regression models, examine associations at the population level, they may overlook meaningful subgroups within heterogeneous populations. A person-centered, cluster-based approach, in contrast, allows for the identification of distinct psychosocial profiles, offering deeper insights into subgroup-specific needs and potential support strategies in hybrid prevention programs [34,35]. Previous research has demonstrated that subtyping based on psychosocial characteristics can enhance intervention design [34].

This study examines the relationship between psychosocial characteristics and eHealth literacy in the context of a hybrid mental health prevention intervention. By identifying distinct psychosocial profiles, it aims to enhance the understanding of eHealth literacy disparities and addresses the following research questions: Can distinct psychosocial profiles be identified through cluster analysis, and how are these profiles associated with eHealth literacy? How do these psychosocial profiles differ in their eHealth literacy levels, and what patterns can be observed? How are sociodemographic variables associated with eHealth literacy?

By systematically analyzing these associations, this study provides insights into how psychosocial characteristics relate to eHealth literacy, contributing to the design of hybrid prevention programs that consider psychosocial diversity to enhance participant engagement and intervention effectiveness.

## Methods

### Study Background

This study was conducted within a project that focuses on developing, piloting, and evaluating an app-supported psychosocial prevention intervention named RV Fit Mental Health. The hybrid secondary prevention intervention consists of an initial 2-week inpatient phase and a 12-week home-based digital training phase supported by a medical app. Medical apps can be either certified medical products or noncertified applications designed to support clinical purposes and health-related functions, such as assisting in diagnostics, therapy, or patient management [36]. The primary aim of the intervention is to improve work ability and psychosocial health in individuals experiencing work-related mental health challenges. The German Federal Ministry of Labor and Social Affairs funds this project. Additional details regarding the project and intervention can be found in the study protocol [37] and are also provided in Multimedia Appendix 1.

### Study Design, Recruitment, and Sample

A quantitative, cross-sectional design was used to investigate the relationship between psychosocial characteristics and eHealth literacy. A total of 324 individuals were invited to participate, of whom 173 participants (53.4%) provided complete data at the beginning of the intervention and were included in the analysis. The intervention was implemented in cohorts, with participants starting at different times between January and December 2024.

Participants were recruited through 2 intervention clinics using diverse materials, including informational flyers, study details, and instructions for web-based data collection. The eligibility criteria were sufficient German language skills for reading and completing study materials, as well as participation in the intervention.

### Data Collection and Measures

Data used for this study were collected during the first 2 days of the initial inpatient phase of the intervention. The survey was administered in German using the secure, web-based platform SoSci Survey. Participation was voluntary; participants could refuse or discontinue participation at any time without consequences. The survey was implemented as a closed format, with access via personalized QR codes or links provided by clinic staff. Responses were entered directly by participants and automatically stored in the secure SoSci Survey database. Each participant created a pseudonym based on a predefined rule, which was used to identify duplicate entries; in such cases, only the first complete response was retained. The final questionnaire included 90 items across 19 pages, with 1-14 items per page depending on content. A back button allowed participants to review and change their responses before submission. The web-based questionnaire was internally tested for usability and technical stability before fielding. An overview of all instruments used for data collection is provided in Table S1 in Multimedia Appendix 2.

eHealth literacy was assessed using the eHealth Literacy and Use Scale (eHLUS), a validated 14-item self-report instrument specifically designed to evaluate digital health literacy in the context of using medical apps [38]. The eHLUS measures 3 dimensions*:* eHealth engagement (4 items), autonomous use and technical access (6 items), and classical eHealth literacy (4 items). A complete listing of all eHLUS items along with their assigned dimensions is provided in Table S2 in Multimedia Appendix 2. The latter dimension includes four items from the eHealth Literacy Scale (eHEALS) [39-41], ensuring conceptual alignment with established measures of eHealth literacy. In addition, eHEALS was used as a complementary measure to assess the ability to locate, evaluate, and use digital health information, providing a basis for direct comparability with previous research. The eHEALS has been frequently used in studies that examined associations with mental health–related outcomes [42-44] and is considered one of the most established instruments in the field of eHealth literacy [45,46]. In contrast, the eHLUS was recently developed and specifically validated among individuals with mental health problems participating in a hybrid digital prevention program [38] but has not yet been widely applied in mental health research. Both instruments were analyzed as continuous measures to preserve metric variability and support a differentiated interpretation of eHealth literacy. Although a cut-off score has been proposed for the eHEALS in earlier studies, no clinically validated threshold exists [47,48]. For the eHLUS, no cut-off scores have been established to date.

Psychosocial characteristics were assessed using validated instruments and categorized into psychosocial burden and resources. Psychosocial burden was measured using the Depression Anxiety Stress Scale with 21 Items (DASS-21), which includes subscales for depression, anxiety, and stress [49,50]. In addition, the Work Ability Index (WAI) was used to assess participants’ self-reported work ability in relation to health and work demands, with lower scores indicating greater psychosocial burden [51].

Psychosocial resources were assessed using the Self-Efficacy, Optimism, Pessimism Scale with 9 Items (SWOP-K9), which captures self-efficacy, optimism, and pessimism as indicators of resilience [52]. Furthermore, Quality of Life (QoL) was measured using the WHOQOL-BREF, which evaluates physical health, psychological health, social relationships, and environmental factors as broader psychosocial resources [53,54].

In addition, sociodemographic data were collected, including age, registered sex (gender), subjective socioeconomic status (SSS) (using the MacArthur Scale) [55,56], and the self-reported number of sick leave days in the past 12 months.

### Statistical Analysis

Statistical analyses were primarily conducted using SPSS statistics (version 29.0 IBM Corp), with Python 3, an open-source programming language, within the open-source Jupyter Notebook Version 7 framework (Project Jupyter) as a supplementary tool for data exploration, visualization, and advanced statistical tasks.

### Cluster Analysis

To identify psychosocial profiles based on characteristics (burdens and resources), we used a data-driven cluster analysis, which uncovers distinct subgroups without prespecifying how these variables interact. In contrast, regression models typically test predefined relationships, making cluster analysis more suitable for capturing psychosocial heterogeneity and its links to eHealth literacy [57-59]. Principal component analysis (PCA) in SPSS was used for dimensionality reduction and to extract key components for K-Means. The included variables were depression, anxiety, and stress (DASS-21); physical, psychological, social, and environmental QoL (WHOQOL-BREF); optimism and self-efficacy (SWOP-K9); and WAI. Pessimism (SWOP-K9) was excluded due to low communality (*h²*=0.264) and weak factor loadings.

To facilitate visualization in a scatterplot and improve pattern recognition, PCA was restricted to 2 dimensions, reducing the complexity of multiple psychosocial variables while preserving key variance for clustering. Although solutions with more than 2 components were initially explored, additional components had eigenvalues below 1.0 (eg, the third component had an eigenvalue of 0.958) and were therefore not retained, following Kaiser criterion. The final 2-component solution explained 60.66% of the total variance and offered a parsimonious and interpretable basis for clustering. The Kaiser-Meyer-Olkin (KMO) measure confirmed suitability adequacy (KMO=0.864), and the Bartlett test of sphericity was significant (*χ*²_45_=816.2; *P*<.001), supporting sufficient intercorrelations for PCA. PCA was performed using principal component extraction with Varimax rotation, ensuring uncorrelated and interpretable components. The rotation converged in 3 iterations, confirming stability. Factor scores were computed via the regression method and stored as PCA_Dim1 and PCA_Dim2 for clustering.

**Figure 1.**
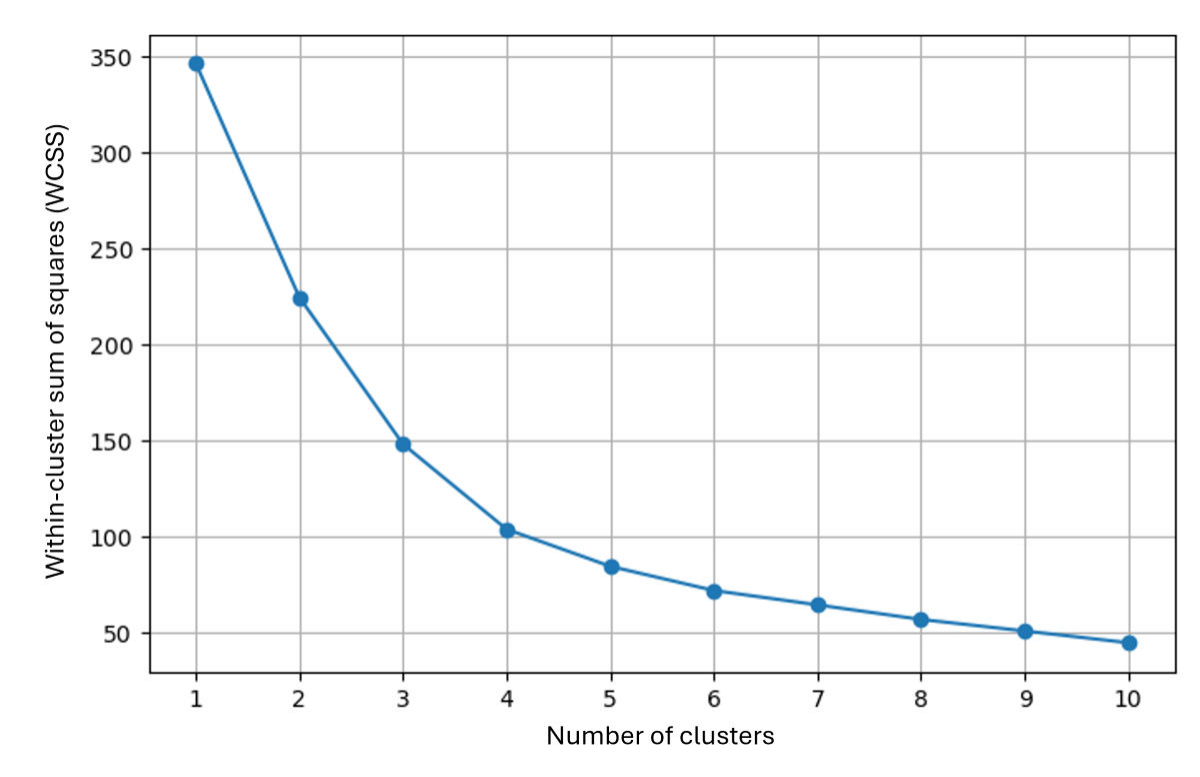
Elbow method for determining the optimal number of clusters.

The Elbow Method in Python determined the optimal number of clusters by evaluating within-cluster sum of squares (WCSS) for solutions from *k*=1 to *k*=10. The elbow point at four clusters (see Figure 1) indicated the best fit. K-Means clustering with four clusters used PCA_Dim1 and PCA_Dim2 as input variables, converging in fewer than 10 iterations. Cluster centers were calculated, and case distribution was recorded.

Exploratory data analysis assessed cluster homogeneity and distribution. Boxplots visualized central tendencies and variability, with means and standard deviations computed per cluster.

#### Internal Validation of the Cluster Solution Using Multivariate Analysis of Variance

To assess the internal validity of the cluster solution, a multivariate analysis of variance (MANOVA) was conducted using the 4-cluster grouping variable (derived from K-means) as the independent variable and the 2 principal components (PCA_Dim1, PCA_Dim2), which served as the input for clustering, as dependent variables (DVs). These components were derived from psychosocial characteristics and represent the underlying structure used to form the clusters. This procedure tested whether the clusters significantly differed along the psychosocial dimensions that initially informed the clustering process.

Prior to MANOVA, assumptions were tested. The Shapiro–Wilk test assessed normality: PCA_Dim1 was normally distributed in clusters 1, 3, and 4 but deviated in cluster 2 (*P*=.03). PCA_Dim2 met normality in clusters 3 and 4 but deviated in clusters 1 (*P*=.04) and 2 (*P*=.005). Despite these moderate violations, MANOVA remained robust as each cluster contained at least 20 participants [60].

Box M test (Box M=13.462, *P*=.16) confirmed homogeneity of variance–covariance matrices. Levene test indicated no significant variance differences for PCA_Dim1 (*P*=.21) or PCA_Dim2 (*P*=.07). Scatterplots showed no major deviations from linearity, and the near-zero correlation minimized multicollinearity risk.

MANOVA was conducted in SPSS using Pillai’s Trace as the primary test statistic due to its robustness against normality violations. Wilks’ λ, Hotelling’s Trace, and Roy’s Largest Root were reported as secondary statistics. Following MANOVA, univariate analysis of variance (ANOVA) identified specific cluster differences, with Tukey’s post hoc tests determining significant pairwise differences.

#### Visualization of the Clusters

A 2-dimensional scatterplot using Python visualized the clusters with PCA_Dim1 and PCA_Dim2 as axes. To illustrate spatial distribution and variability, 95% confidence ellipses were calculated from the covariance matrix and overlaid on the plot, enabling the assessment of potential overlaps. This visualization complemented statistical analyses, such as MANOVA, by highlighting cluster separation and internal variability.

Cluster-specific boxplots were used to visualize the distributions of psychosocial characteristics, with *z* scores standardizing the data for comparability. Negative variables were inverted to ensure that higher scores consistently represented positive outcomes.

Bar and spider charts visualize cluster-specific mean *z* scores to highlight multidimensional differences in eHealth literacy. The 5 analyzed eHealth literacy variables are normalized using *z* scores to ensure comparability.

### Descriptive Analysis

In addition to clustering, the psychosocial characteristics included in PCA were analyzed descriptively for each cluster. This involved calculating means and 95% CIs for PCA variables along with pessimism, which was excluded from PCA but retained for further insights. This step enhanced the interpretation and practical relevance of cluster differences.

### Correlation Analysis

Spearman rank correlation examined associations between eHealth literacy, psychosocial characteristics, and sociodemographic variables. This nonparametric test accounted for ordinal variables and potential nonnormal distributions. The analysis included the 3 eHLUS dimensions as DVs, while psychosocial characteristics, sociodemographic variables such as age and SSS*,* and behavioral measures such as the number of sick leave days served as independent variables.

The correlations were calculated using SPSS. Statistical significance was evaluated at a 2-sided alpha level of .05, with results adjusted for multiple comparisons using Bonferroni correction where necessary. Correlation coefficients (r values) were interpreted based on established thresholds for effect sizes: small (*r*=0.10‐0.29), moderate (*r*=0.30‐0.49), and strong (*r*≥0.50).

### General Linear Model Analysis

A General Linear Model (GLM) was applied to investigate the association of cluster membership and covariates on eHealth literacy. To improve the distributional properties and stabilize variances of the DVs, transformations were applied: Box-Cox transformations for eHEALS total score and eHLUS dimension eHealth engagement, and square-root transformations for the remaining DVs (eHLUS total score, eHLUS dimensions: eHealth literacy; autonomous use and technical access). Cluster membership was included as a fixed factor, while age (metric) and SSS (ordinal) were included as covariates due to their significant correlations with the DVs. Gender was excluded as it did not show any significant relationship with the DVs (*P*>.05). Statistical significance was set at *α*=.05, and adjusted *R*² values ranged from 0.053 for eHealth engagement to 0.230 for autonomous use and technical access, indicating moderate explanatory power for certain DVs. Residual normality was assessed using the Kolmogorov-Smirnov and Shapiro-Wilk tests, with 4 out of 5 residuals meeting the assumption (*P*>.05). Only the eHLUS dimension eHealth literacy showed a marginal deviation (*P*=.04), which was deemed acceptable based on Q-Q plots. Homoscedasticity was confirmed, as Levene test indicated no significant differences in error variances across clusters (*P*>.05), and Box M test verified the homogeneity of covariance matrices (Box M=56.539; *P*=.19). Scatterplots demonstrated linearity, which showed consistent relationships between the DVs and covariates. Correlations among covariates were weak (*r*<0.10; *P*>.05), ruling out multicollinearity concerns.

As implemented in SPSS, the GLM procedure included both a MANOVA to assess the overall effect of cluster membership, age, and SSS across all eHealth literacy variables simultaneously, and a subsequent ANOVA to examine effects on each variable separately. This combined analytical strategy was applied to enable a comprehensive assessment of group differences and covariate associations within a unified model structure.

### Ethical Considerations

The study was approved by the Ethics Committee of the TUM School of Medicine and Health (approval number 2023‐316 S-SB) and conducted in accordance with institutional, national, and international regulations as well as the Declaration of Helsinki, including requirements regarding the protection of personal information, privacy, and human rights*.* Participants were adults (21-65 years), including both men and women; no vulnerable populations (eg, minors, patients unable to provide consent) were involved. All participants provided informed consent via a web-based form after receiving comprehensive study information. This included details on the purpose of the study, the estimated duration of the survey (approximately 15‐20 minutes), the responsible investigators, the voluntary nature of participation, their right to withdraw at any time without consequences, and data handling procedures. Data were pseudonymized and handled confidentially in compliance with the General Data Protection Regulation. No personally identifiable information (such as names, email addresses, or IP addresses) was collected, and no identifiable features of participants will be published. Data were initially stored in the secure SoSci Survey database and subsequently transferred to secure servers at the Technical University of Munich, where they will be retained for up to 10 years. Participants received a fixed financial compensation for their participation, which was administered on-site by the cooperating clinics.

## Results

### Overview

Data from 173 participants were analyzed, with a mean age of 50.9 years (SD 10.3; Min=21, Max=65). Among them, 72.8% identified as female, and 27.2% identified as male. The mean SSS, measured on a scale from 1 (lowest) to 10 (highest), was 5.6 (SD 1.6; Min=2; Max=9), considered moderate. Cumulative sick leave days over the past 12 months were reported by 171 individuals, with a mean of 46.9 days (SD 55.1; Min 0, Max 365), notably exceeding the national average of 14.8 days per employee in 2024 [61], which reflects the elevated health burden among participants of a prevention program targeting mental health. All subsequent analyses were conducted using data from 173 participants.

### Dimensionality Reduction of Psychosocial Determinants

The PCA successfully reduced the dataset to 2 dimensions, which explained 60.66% of the total variance together. Component 1 (eigenvalue: 3.391) accounted for 39.31% of the variance, while Component 2 (eigenvalue: 2.135) accounted for 21.35%. The rotated component matrix revealed transparent and interpretable variable loading patterns (see Table 1). Component 1 includes psychosocial characteristics with high positive loadings for self-efficacy (0.774), optimism (0.752), psychological QoL (0.684), social QoL (0.703), and environmental QoL (0.531), representing psychosocial resources. The negative loadings for depression (−0.799), anxiety (−0.623), and stress (−0.665) on Component 1 suggest that participants with higher psychosocial resources also tend to report lower psychosocial burden. Component 2 captured work-related and physical health factors, with high loadings for WAI (0.845) as an indicator of psychosocial burden and physical QoL (0.762) as a resource contributing to overall well-being. The commonalities of the variables ranged between 0.437 and 0.761, indicating that most variables were well-explained by the 2 extracted components. The lowest communality was observed for the QoL environment (0.437), which remained within the acceptable range (≥0.4) [62].

**Table 1.** Factor loadings of the principal component analysis.

Variable	Component 1	Component 2
Self-efficacy	0.774**^a^**	0.174
Optimism	0.752^a^	0.243
Psychological QoL^b^	0.684^a^	0.400
Social QoL	0.703^a^	−0.172
Environmental QoL	0.531^a^	0.394
Depression	−0.799^a^	−0.350
Anxiety	−0.623^a^	−0.348
Stress	−0.665^a^	−0.403
WAI^c^	0.126	0.845^a^
Physical QoL	0.196	0.762^a^

a These values indicate the stronger loading of a variable on the respective component.

b QoL: Quality of Life

c WAI: Work Ability Index

### Optimal Number of Clusters for Psychosocial Profiles

**Figure 2.**
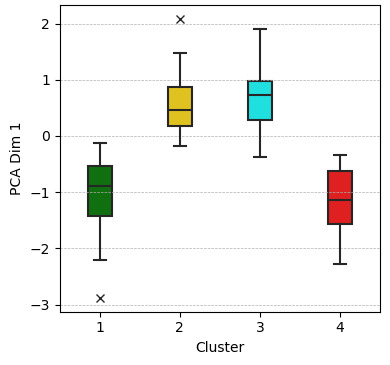
Boxplot of PCA_Dim1 by cluster. PCA Dim 1: principal component analysis, dimension 1.

**Figure 3.**
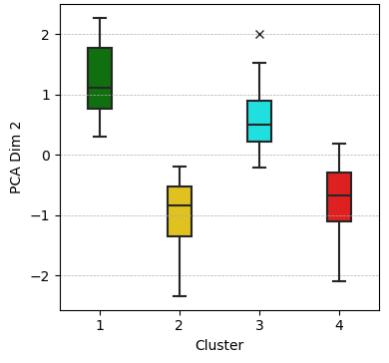
Boxplot of PCA_Dim2 by cluster. PCA Dim 2: principal component analysis, dimension 2.

The Elbow Method revealed a clear “elbow point” at 4 clusters, indicating an effective balance between intracluster homogeneity and intercluster differentiation (Figure 1). Based on this result, K-Means clustering with four clusters was performed using PCA_Dim1 and PCA_Dim2 as input variables, converging after nine iterations. The resulting cluster centers, along with the means and SDs of the PCA dimensions, are presented in Table 2. In addition, an exploratory boxplot analysis was conducted to assess cluster homogeneity and distribution. Boxplots for PCA_Dim1 (Figure 2) and PCA_Dim2 (Figure 3) visualized central tendencies and variability within each cluster, confirming distinct distributions.

**Table 2.** Cluster centers of the final solution (K-Means analysis).

Cluster	Cases per cluster	PCA_Dim1, mean (SD)	PCA_Dim2, mean (SD)
1	27	−1.023 (0.71)	1.225 (0.62)
2	45	0.538 (0.51)	−0.974 (0.60)
3	65	0.689 (0.52)	0.559 (0.47)
4	36	−1.148 (0.56)	−0.710 (0.56)

### Cluster Validation, MANOVA, and Post Hoc Analyses

The MANOVA confirmed significant differences for the effect of cluster among the 4 clusters in the 2 principal components (Pillai’s Trace=1.4021, Wilks’ λ=0.0890, Hotelling’s Trace=4.7163, Roy’s Largest Root=2.5688; *F*_6,336_=131.69, *P*<.001).

ANOVA demonstrated significant differences for PCA dimensions (PCA_Dim1: *F*_3, 169_=128.51, *P*<.001; *PCA_Dim2: F*_3, 169_=135.83, *P*<.001). Tukey’s post hoc tests revealed significant differences between most cluster pairs (*P*<.05), except for clusters 1 and 4, as well as 2 and 3 in PCA_Dim1, and clusters 2 and 4 in PCA_Dim2. These findings indicate that, while the MANOVA revealed significant overall differences between the clusters, not all pairwise comparisons reached significance in the post hoc tests.

### Description of the Clusters

The 4 identified psychosocial characteristic profiles, each with varying levels of eHealth literacy, are described below. To improve clarity, the most contrasting clusters (3 and 4) are presented first, followed by the remaining groups (1 and 2), with a focus on key differences. Detailed statistics can be found in Table 3 (eHealth literacy) and Table 4 (psychosocial characteristics).

**Table 3.** Descriptive statistics of eHealth literacy variables by cluster.

Variable	Cluster 1, mean (95%CI)	Cluster 2, mean (95%CI)	Cluster 3, mean (95%CI)	Cluster 4, mean (95%CI)
eHEALS^a^ total score	30.78 (28.34‐33.21)	29.51 (27.55‐31.48)	32.55 (31.18‐33.93)	31.14 (29.21‐33.07)
eHLUS^b^ total score	53.81 (50.00‐57.63)	50.87 (48.42‐53.31)	56.48 (54.39‐58.56)	53.03 (49.74‐56.31)
Dimension: eHealth engagement	13.74 (12.30‐15.18)	13.02 (12.04‐14.00)	14.55 (13.74‐15.37)	13.89 (12.74‐15.04)
Dimension: autonomous use and technical access	24.04 (22.28‐25.79)	22.42 (21.15‐23.70)	25.29 (24.25‐26.33)	23.39 (21.74‐25.04)
Dimension: eHealth literacy	16.04 (14.97‐17.10)	15.42 (14.61‐16.23)	16.63 (15.95‐17.31)	15.75 (14.69‐16.81)

a eHEALS: eHealth Literacy Scale.

b eHLUS: eHealth Literacy and Use Scale.

**Table 4.** Descriptive statistics of psychosocial outcomes by cluster.

Variable	Cluster 1, mean (95%CI)	Cluster 2, mean (95%CI)	Cluster 3, mean (95%CI)	Cluster 4, mean (95%CI)
Depression	9.78 (8.20‐11.36)	7.51 (6.58‐8.44)	4.20 (3.57‐4.83)	13.33 (12.17‐14.49)
Anxiety	5.89 (4.45‐7.32)	5.04 (4.17‐5.91)	2.72 (2.06‐3.38)	8.94 (7.71‐10.18)
Stress	11.56 (9.98‐13.13)	9.98 (9.13‐10.83)	6.88 (6.21‐7.54)	14.72 (13.61‐15.83)
QoL^a^ Physical	55.16 (52.79‐57.53)	42.46 (40.35‐44.57)	54.84 (53.19‐56.48)	40.97 (38.75‐43.19)
QoL Psychological	56.79 (52.15‐61.43)	56.67 (54.00‐59.33)	64.29 (62.29‐66.30)	44.10 (41.07‐47.12)
QoL Social	37.96 (31.19‐44.73)	68.70 (63.69‐73.71)	68.08 (64.24‐71.91)	45.14 (39.53‐50.75)
QoL Environment	70.37 (65.96‐74.79)	69.38 (66.32‐72.43)	75.38 (73.09‐77.68)	58.07 (54.21‐61.93)
Self-efficacy	2.17 (2.00‐2.34)	2.64 (2.54‐2.75)	2.86 (2.75‐2.96)	1.98 (1.86‐2.10)
Optimism	2.20 (1.96‐2.45)	2.62 (2.48‐2.77)	2.98 (2.85‐3.10)	1.81 (1.62‐1.99)
WAI^b^	34.26 (32.27‐36.25)	21.71 (19.95‐23.47)	31.83 (30.67‐32.99)	22.83 (21.00‐24.67)

a QoL: Quality of Life.

b WAI: Work Ability Index.

#### Cluster 3 (n=65)

This cluster exhibits the most favorable psychosocial characteristics and the highest eHealth literacy levels among all clusters. Participants show the strongest psychosocial resources, with the highest levels of self-efficacy and optimism. Their reported QoL is also the highest across all dimensions. At the same time, their psychosocial burden appears to be the lowest, with the lowest levels of depression, anxiety, and stress. Their work ability is the second highest among all clusters, suggesting a relatively strong perceived capacity to meet health and work demands.

These psychosocial characteristics coincide with the highest eHealth literacy levels across all measured dimensions and clusters.

#### Cluster 4 (n=36)

This cluster exhibits the least favorable psychosocial characteristics among all clusters while demonstrating moderate eHealth literacy levels. Participants report the lowest psychosocial resources, with self-efficacy and optimism at the lowest levels. Their QoL is the lowest across most dimensions, except for social QoL, which is slightly higher than in Cluster 1. At the same time, their psychosocial burden is the highest among all clusters, with elevated levels of depression, anxiety, and stress. Their work ability is low and only marginally higher than in Cluster 2.

Despite these challenges, participants in this cluster demonstrate mid-range eHealth literacy levels, similar to Cluster 1 and slightly higher than those in Cluster 2. While their ability to engage with and use digital health resources is not as pronounced as in Cluster 3, their competencies remain stable.

#### Cluster 1 (n=27)

This cluster exhibits a complex psychosocial characteristics profile and moderately favorable eHealth literacy levels. Participants demonstrate mixed psychosocial resources. Their self-efficacy and optimism are the second lowest among all clusters. Regarding QoL, they report the highest physical QoL, mid-range psychological and environmental QoL, and the lowest social QoL. At the same time, their psychosocial burden is elevated but not uniformly high. They exhibit the second-highest levels of depression, anxiety, and stress, surpassed only by Cluster 4. Their WAI is the highest among all clusters.

Their eHealth literacy levels are moderately favorable, ranking above Cluster 2 but below Cluster 3. While their engagement with digital health tools and their ability to use them autonomously is limited compared to the highest-performing cluster, they do not exhibit substantial barriers in using eHealth resources.

#### Cluster 2 (n=45)

This cluster exhibits a mixed psychosocial characteristics profile and the lowest eHealth literacy levels among all clusters. Participants demonstrate relatively strong psychosocial resources, with self-efficacy and optimism ranking second-highest among all clusters. Their social QoL is the highest, indicating strong interpersonal connections. In contrast, physical QoL is low, while psychological and environmental QoL fall within the mid-range, reflecting a more nuanced profile of well-being. At the same time, their psychosocial burden is moderate. Their work ability is the second lowest, reflecting a perceived strain in balancing health and work demands. Depression, anxiety, and stress levels fall within the mid-range, indicating a moderate psychosocial burden. Their eHealth literacy is the lowest among all clusters, with the lowest levels of eHealth engagement, autonomous use, and technical access.

### Cluster Visualization: Psychosocial Characteristics

**Figure 4.**
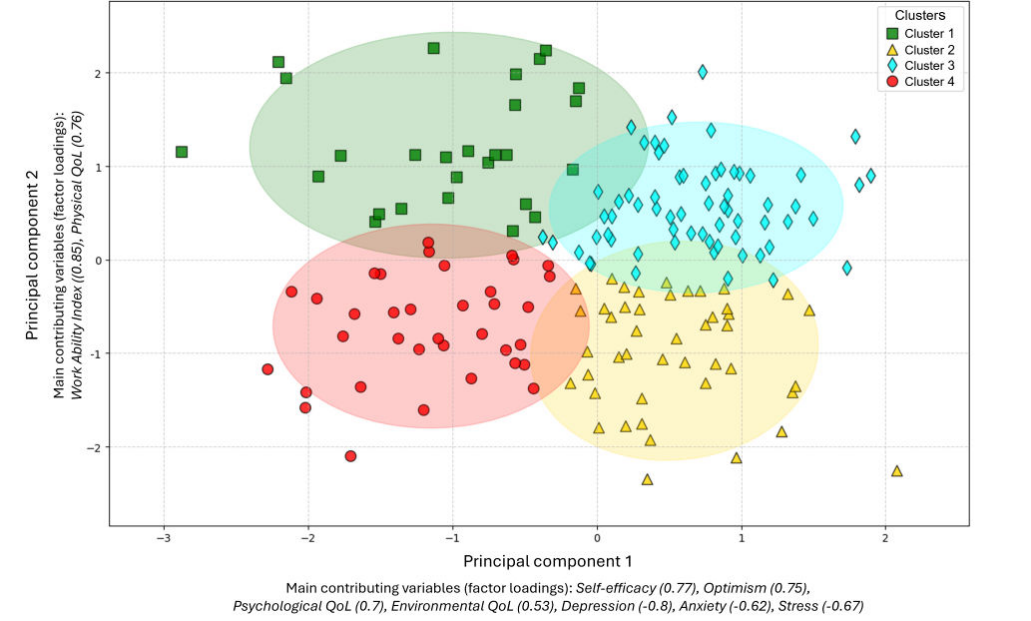
Scatterplot of the clusters on PCA dimensions.

The scatterplot (Figure 4) illustrates the distribution of the four clusters across the PCA dimensions, accompanied by 95% confidence ellipses. These ellipses indicate slight overlaps between adjacent clusters along both the x- and y-axis. Cluster 1 (green squares) overlaps moderately with Clusters 3 (cyan diamonds) and 4 (red circles), while Cluster 2 (yellow triangles) shows similar overlap with Clusters 3 and 4. Likewise, Cluster 3 overlaps with Clusters 1 and 2, and Cluster 4 overlaps with Clusters 1 and 2. These overlaps suggest shared characteristics between neighboring clusters while maintaining distinct separations. The variability within the clusters is reflected in the sizes of the ellipses. Cluster 1 has the largest ellipse, indicating greater variability within this group, followed by Cluster 4. Clusters 2 and 3 have smaller ellipses, suggesting more compact groupings.

**Figure 5.**
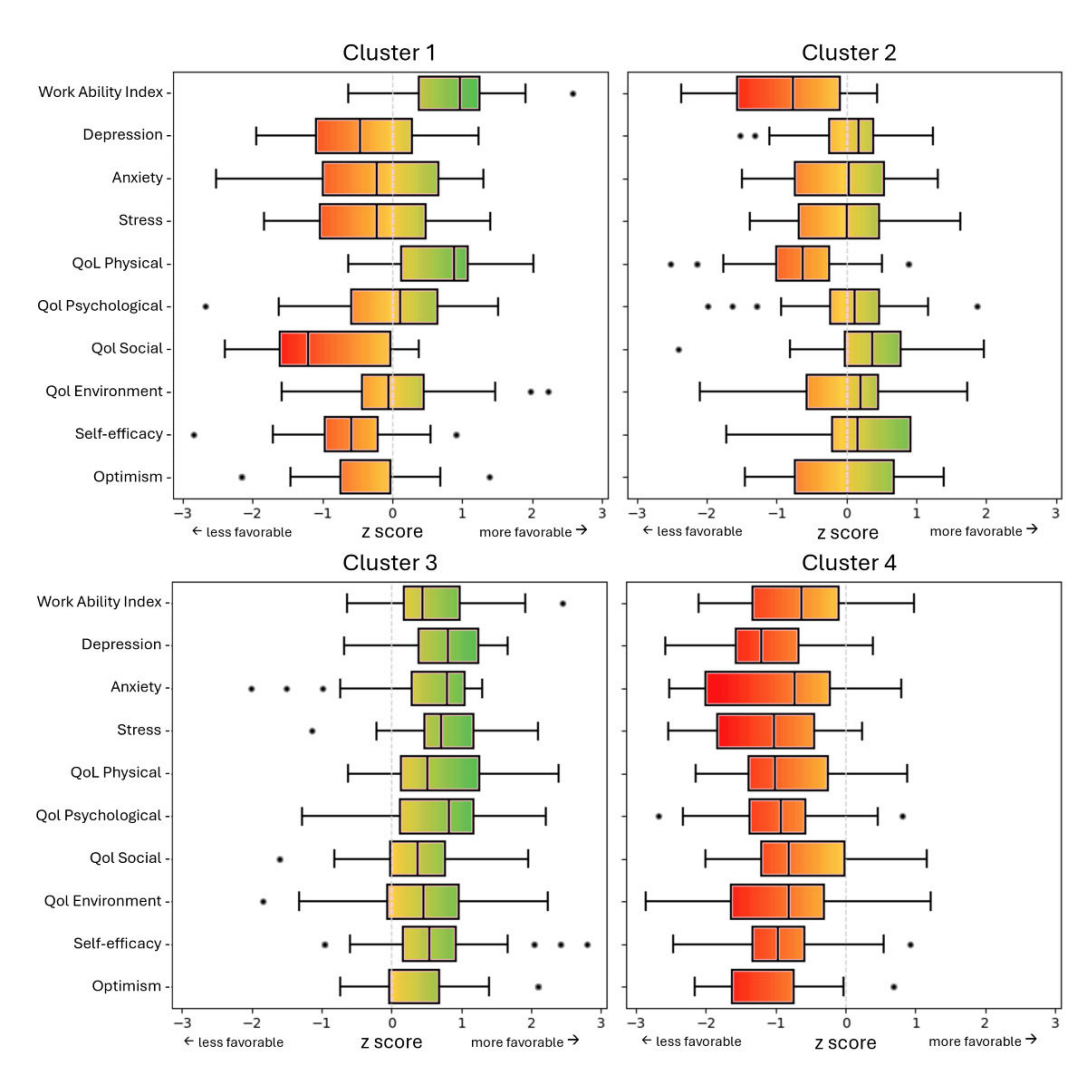
Color-coded z score profiles across clusters: relative differences in psychosocial characteristics.

The color-coded boxplots (Figure 5) illustrate the psychosocial characteristic profiles across clusters, using a gradient from red (less favorable outcomes) to green (more favorable outcomes). These colors represent relative differences between clusters rather than absolute measures of health or illness.

Cluster 3 consistently exhibits the most favorable psychosocial characteristics (green hues), whereas Cluster 4 shows the least favorable characteristics (orange and red shades). Clusters 1 and 2 display mixed profiles: Cluster 1 combines moderate WAI (green) with limitations in social QoL and self-efficacy (orange), while Cluster 2 contrasts strong social QoL (green) with lower physical QoL and WAI (orange to red).

### Cluster Visualization: eHealth Literacy

**Figure 6.**
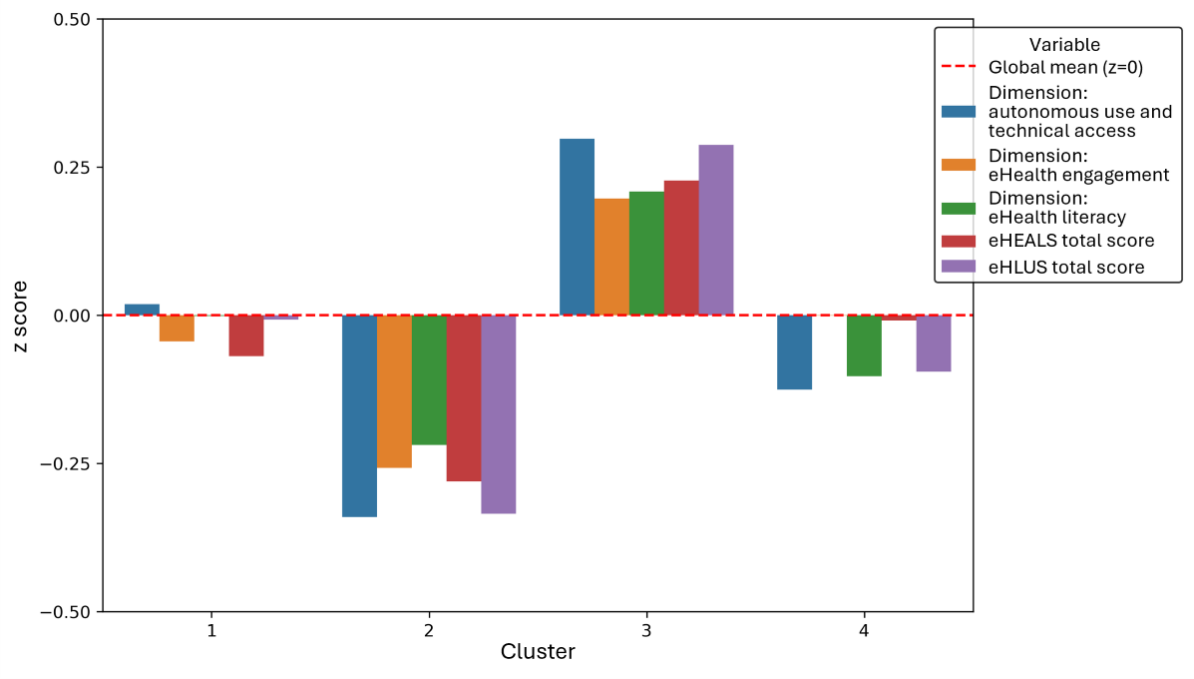
Bar chart of *z* scores for eHealth literacy dimensions and total scores across clusters. eHEALS: eHealth Literacy Scale; eHLUS: eHealth Literacy and Use Scale.

**Figure 7.**
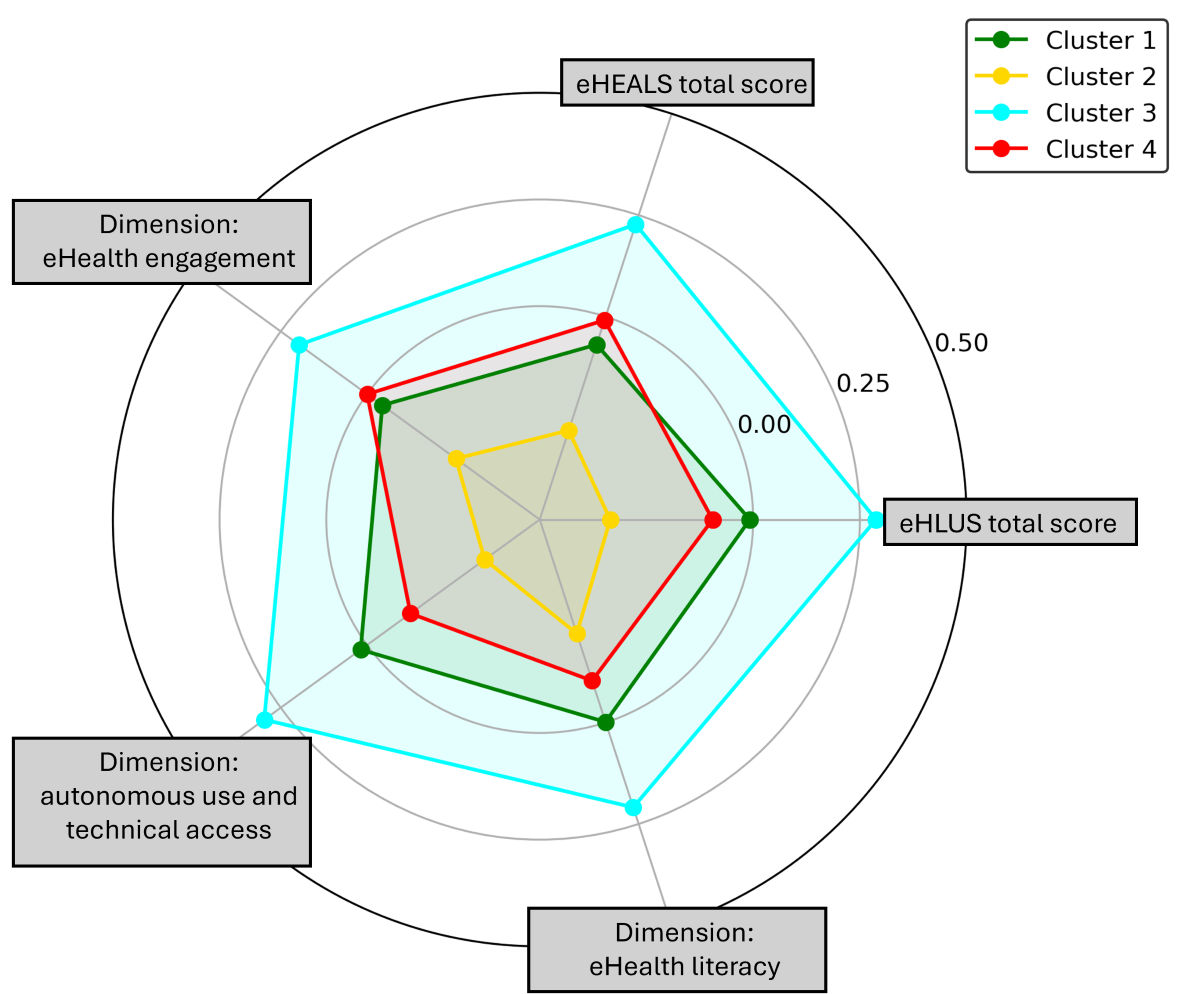
Spider chart of *z* scores for eHealth literacy dimensions and total scores across clusters. eHEALS: eHealth Literacy Scale; eHLUS: eHealth Literacy and Use Scale.

Figure 6 displays bar charts of average *z* scores for five eHealth literacy variables per cluster. Cluster 3 consistently shows above-average scores, reflecting high eHealth literacy, while Cluster 2 demonstrates negative z scores, indicating lower competencies. Clusters 1 and 4 lie in between, with moderate scores that vary across variables. To complement this visualization, Figure 7 presents a spider chart, offering a multidimensional perspective on these patterns. The results were robust, even without the removal of outliers, confirming their minimal impact.

### Correlation Analysis of eHealth Literacy Dimensions

The correlation analysis (see Table 5) revealed significant associations between eHealth literacy dimensions (eHealth engagement, autonomous use and technical access*,* and eHealth literacy), psychosocial characteristics, as well as age, sick leave days*,* and SSS*.*

**Table 5.** Correlation coefficients between eHealth literacy dimensions and psychosocial variables.

Variable	eHealth engagement		Autonomous use and technical access		eHealth literacy	
	r^a^	*P value*	*r*	*P* value	*r*	*P* value
Depression	−0.061	.43	−0.142	.06	−0.121	.11
Anxiety	−0.001	.99	−0.133	.08	−0.065	.39
Stress	0.014	.85	−0.025	.75	−0.002	.98
QoL^b^ Physical	0.111	.15	0.102	.18	0.094	.22
QoL Psychological	−0.021	.78	0.101	.18	0.099	.20
QoL Social	0.071	.35	0.091	.23	0.135	.08
QoL Environment	0.209	.006	0.382	<.001	0.386	<.001
Self-efficacy	0.103	.18	0.382	<.001	0.386	<.001
Optimism	0.138	.07	0.141	.06	0.198	.009
Pessimism	0.090	.24	−0.153	.045	−0.117	.13
WAI^c^	0.138	.07	0.191	.01	0.169	.03
Age	−0.190	.01	−0.025	.74	−0.063	.42
Sick leave days	−0.080	.30	−0.338	<.001	−0.278	<.001
SSS^d^	0.143	.06	0.222	.003	0.240	.001

a *r*: Pearson correlation coefficient.

b QoL: Quality of Life.

c WAI: Work Ability Index.

d SSS: subjective socioeconomic status.

For the eHLUS dimension eHealth engagement, significant positive correlations were observed with environmental QoL (*r*=0.209; *P*=.006), indicating that individuals with higher engagement in eHealth literacy tend to report greater satisfaction with their physical and social surroundings. In addition, a weak negative correlation with age (*r*=−0.190; *P*=.01) suggests that younger individuals are more engaged with eHealth tools. However, no significant correlations were found between eHealth engagement and other variables.

eHLUS dimension autonomous use and technical access demonstrated moderate positive correlations with self-efficacy (*r*=0.382; *P*<.001) and environmental QoL (*r*=0.382; *P*<.001), suggesting that individuals with greater confidence in their abilities and satisfaction with their environment are more likely to use eHealth tools autonomously. This dimension also exhibited small correlations with SSS (*r*=0.222; *P*=.003) and WAI (*r*=0.191; *P*=.01). Negative correlations were found with sick leave days due to illness (*r*=−0.338; *P*<.001) and pessimism (*r*=−0.153; *P*=.05), indicating that individuals with fewer psychosocial challenges and health disruptions are more likely to use eHealth tools independently.

eHLUS dimension eHealth literacy showed a similar pattern of associations, with significant positive correlations with self-efficacy (*r*=0.386; *P*<.001), optimism (*r*=0.198; *P*=.009), environmental QoL (*r*=0.386; *P*<.001), SSS (*r*=0.240; *P*=.001), and WAI (*r*=0.169; *P*=.03). Negative correlations were observed with sick leave days (*r*=−0.278 *P*<.001), indicating that individuals with fewer health-related disruptions exhibit higher levels of eHealth literacy.

### GLM Results: Associations Between Clusters, Covariates, and eHealth Literacy

The GLM examined the associations between eHealth literacy dimensions and cluster membership, age, and SSS (see Table S1 in Multimedia Appendix 3). Significant associations were found for several predictors across the DVs, although some did not reach statistical significance.

Age was significantly associated with all eHealth literacy dimensions (*P* values ranging from <.001 to .01), with younger individuals consistently reporting higher eHealth literacy levels. SSS was significantly associated with the eHEALS total score (*P*=.03), the eHLUS total score (*P*=.004), and the eHLUS dimensions autonomous use and technical access (*P*=.01) and eHealth literacy (*P*=.001). Participants with a higher SSS level tended to report higher eHealth literacy in these dimensions.

Cluster membership was significantly associated with the eHLUS total score (*P*=.01) and the eHLUS dimension autonomous use and technical access (*P*=.004). Post hoc analyses using the Tukey test identified significant differences between clusters, specifically between Cluster 3 and Cluster 2, with Cluster 3 showing significantly higher values than Cluster 2 for both the eHLUS total score and the eHLUS dimension autonomous use and technical access (*P*=.004, *P*=.003).

The model explained the highest variance for the eHLUS dimension autonomous use and technical access (adjusted *R*²=0.230) and the eHLUS total score (adjusted *R*²=0.202). A lower proportion of variance was explained for the eHLUS dimension eHealth engagement (adjusted *R*²=0.053), indicating a weaker model fit for this variable. Multivariate tests confirmed the robustness of the findings; detailed results are provided in Table S2 in Multimedia Appendix 3.

## Discussion

### Principal Findings

The findings of this study highlight the complex interplay between psychosocial characteristics and eHealth literacy within a hybrid secondary prevention program for mental health. Cluster analysis enabled a nuanced examination of how psychosocial burdens and resources relate to eHealth literacy.

Four distinct psychosocial profiles were identified, indicating that psychosocial characteristics are associated with eHealth literacy, though patterns of association vary across dimensions. Significant differences based on cluster membership emerged in the overall eHLUS score and the eHLUS dimension of autonomous use and technical access. No significant associations were found for the overall eHEALS score and the eHLUS dimensions eHealth engagement and eHealth literacy.

To further interpret these findings, the psychosocial characteristics of the clusters were examined in relation to their eHealth literacy levels. Cluster 3, which exhibited the most favorable psychosocial profile, combining high psychosocial resources and low burden, also demonstrated the highest eHealth literacy across all dimensions. This pattern may reflect a resilient subgroup with minimal barriers to digital engagement. In contrast, Cluster 4, despite having the least favorable psychosocial characteristics overall, showed mid-range eHealth literacy levels, challenging the assumption that less favorable psychosocial characteristics necessarily correspond to the lowest eHealth literacy. This nonlinear pattern is also evident in Cluster 2, which displayed the lowest eHealth literacy levels despite an overall moderate psychosocial profile. While participants in this cluster reported relatively poor work ability and physical QoL, they also had one of the highest social QoL scores. In fact, when considering mean scores across all psychosocial characteristics, Cluster 2 appeared more balanced than Cluster 1, which exhibited both psychosocial characteristics and eHealth literacy at moderate levels. The results emphasize that eHealth literacy is not uniformly distributed across psychosocial profiles. Rather than following a straightforward gradient, our findings indicate a complex relationship between specific psychosocial characteristics and eHealth literacy. While traditional models suggest a linear relationship where higher psychological burden corresponds to lower eHealth literacy, our approach reveals a more nuanced and heterogeneous pattern. Even when examined in isolation, no single psychosocial characteristic variable visually indicates a clear linear trend with eHealth literacy, reinforcing the need for a more nuanced understanding of this relationship.

In the correlation analysis of isolated psychosocial and sociodemographic variables, QoL environment was the only variable to show a significant correlation across all three eHLUS dimensions. Self-efficacy and work ability were significantly correlated with the eHLUS dimensions autonomous use and technical access as well as eHealth literacy. In addition, optimism and pessimism each correlated with only one of the eHLUS dimensions. Overall, these findings suggest that eHealth literacy may be more closely linked to psychosocial resources, particularly resilience factors, than to psychosocial burdens.

Regarding sociodemographic variables, correlation analyses showed that fewer sick leave days were associated with higher scores in both autonomous use and technical access and eHealth literacy. Further, the GLM revealed that age was significantly associated with all eHealth literacy dimensions, with younger individuals consistently demonstrating higher eHealth literacy levels. SSS also had a significant effect on the overall eHEALS and eHLUS scores, as well as on the eHLUS dimensions autonomous use, technical access, and eHealth literacy, indicating that participants with higher SSS tended to report higher eHealth literacy. Notably, gender did not show a significant relationship with eHealth literacy in our analysis.

The inclusion of age and SSS as covariates reflects the relevance of social determinants for variation in eHealth literacy. These factors represent broader structural conditions that may also relate to psychosocial characteristics. For instance, financial strain or job insecurity as reflected in lower SSS, may coincide with increased stress or reduced self-efficacy. Although not explicitly examined, such interdependencies may help contextualize subgroup differences in digital health engagement. These findings emphasize the importance of considering both psychosocial and social determinants of health when designing hybrid secondary prevention programs.

Examining these psychosocial subgroups is critical for tailoring hybrid interventions, as varying psychosocial burdens may lead to distinct challenges in behavioral adaptation and sustained app use. These challenges likely differ across participant subgroups, requiring targeted strategies at different intervention stages (in-person or digital) that account for differences in digital engagement and self-management skills. Health care professionals play a crucial role in this process by providing tailored support that meets the specific needs of each subgroup. Their involvement ensures that participants receive optimal guidance to navigate both the digital and in-person components of hybrid interventions effectively, addressing subgroup-specific barriers.

Implementing such individualized support in routine health care settings presents several challenges. Health care professionals vary in their eHealth literacy and often lack adequate support, training opportunities, and resources to provide individualized digital guidance during regular consultations [63,64]. To address these barriers, the integration of digital health navigators or trained support personnel could offer valuable assistance, particularly for participants with lower eHealth literacy [65]. These roles can facilitate meaningful engagement through structured onboarding, low-threshold support, and continuous accompaniment. Rather than excluding individuals with limited digital competencies from online treatment options, tailored support can promote their participation and ensure equitable access [65].

In this context, stratifying digital health interventions based on the combination of psychosocial profiles and eHealth literacy levels identified through clustering offers a practical framework for adapting support intensity, interface complexity, and onboarding formats to individual needs. These tailored implementation strategies acknowledge the heterogeneity of user prerequisites and lay the foundation for more equitable and responsive hybrid prevention programs. By integrating these insights into intervention design, hybrid prevention programs can be adapted to overcome subgroup-specific barriers, enhance eHealth engagement, and improve accessibility. In addition, these findings help health care professionals better understand the challenges different subgroups face in adopting eHealth solutions, enabling them to provide more targeted support.

### Comparison With Prior Work

Prior studies have shown that higher eHealth literacy is linked to lower psychosocial burden. Yang et al [66] found significant negative correlations between eHealth literacy and depression, insomnia, and post-traumatic stress disorder, while Akingbade et al [67] reported a reduced likelihood of anxiety and depression among individuals with high eHealth literacy. Our findings partially align with this, as we observed that psychosocial resources (eg, self-efficacy and optimism) were positively associated with eHealth literacy. However, unlike some prior studies, we did not find a direct association between psychosocial burden (eg, depression and stress) and eHealth literacy, suggesting that digital engagement may be more closely related to resilience factors than distress.

Sociodemographic disparities in eHealth literacy are widely documented, particularly regarding age and education. Consistent with prior research, we found that younger individuals and those with higher SSS exhibited significantly higher eHealth literacy. These results align with broader findings that eHealth literacy declines with age and is positively associated with education and income [68,69]. While gender differences in eHealth literacy have been inconsistently reported in the literature, our analysis found no significant gender effect, which supports findings from previous large-scale studies that suggest gender is not a primary determinant once socioeconomic factors are controlled [68].

Much of the existing research on eHealth literacy has relied on variable-centered methods (eg, correlations and regressions), linking eHealth literacy to factors like depression, age, and education [67,69]. In contrast, our study applied cluster analysis to identify distinct subgroups, capturing heterogeneity in eHealth literacy, psychosocial burden, and resources. This approach aligns with prior work demonstrating that clustering can reveal meaningful subpopulations. For example, Petrič et al [46] identified eHealth literacy-based user segments in online health communities, distinguishing highly engaged help-seekers from low-engagement users. Similarly, Andersen et al [70] profiled hospital patients, isolating a vulnerable subgroup with low eHealth literacy, older age, and lower education. These findings underscore how person-centered approaches offer deeper insights into eHealth literacy disparities, informing targeted interventions.

### Limitations

This study has several limitations that warrant consideration. The cross-sectional design restricts the ability to draw causal inferences between psychosocial characteristics and eHealth literacy. While this limits our understanding of temporal or directional effects, the cross-sectional nature is sufficient to identify associations and patterns that provide a strong foundation for future longitudinal studies. The homogeneity of the sample, which included participants with adequate German language proficiency and involvement in the RV Fit Mental Health intervention, may constrain the generalizability of the findings to other populations. However, this focus on a specific, well-defined group ensures internal validity and allows targeted insights into individuals’ psychosocial and eHealth needs in hybrid secondary prevention programs. The recruitment process and eligibility criteria introduce the possibility of selection bias, as only participants who were actively engaged in the intervention and met language proficiency requirements were included. This could mean the sample reflects a more motivated and health-conscious group than the general population. Nonetheless, this targeted recruitment aligns with the study’s aim to understand eHealth literacy within the context of a specific intervention, making the sample highly relevant to the research objectives. In addition, both the eHEALS and eHLUS are self-report instruments that reflect participants’ perceived abilities and motivational orientation toward digital health use, rather than objectively tested eHealth competencies. While both instruments provide valuable continuous scores, the resulting scores lack validated cutoffs and therefore cannot be translated into clinically actionable categories. Furthermore, potential interrelations between psychosocial characteristics and social determinants of health were not explicitly modeled, as the primary aim was to identify psychosocial subgroups. Including additional structural variables may have compromised model parsimony, given the sample size and the exploratory focus of this study. These considerations underline the importance of interpreting the results within their methodological context while affirming the study’s strengths in exploring the interplay between psychosocial characteristics and eHealth literacy.

### Conclusions

This study highlights the importance of a person-centered approach in understanding the interplay between psychosocial characteristics and eHealth literacy within a hybrid secondary prevention program. By using cluster analysis, we identified distinct psychosocial subgroups with varying levels of eHealth literacy, which would not have been fully captured through traditional variable-centered methods. This analytical approach allowed us to uncover nuanced relationships, demonstrating that psychosocial resources, rather than psychosocial burden alone, are closely linked to eHealth literacy. Our findings emphasize that eHealth literacy is not uniformly distributed and that subgroup-specific differences must be considered when designing and implementing eHealth interventions.

The implications of these findings for hybrid prevention programs are substantial. Health care professionals play a critical role in supporting participants by tailoring interventions to match individual needs. By addressing both eHealth literacy deficits and psychosocial challenges, personalized support strategies can enhance the effective adoption and sustained use of eHealth tools. This targeted approach may improve user engagement, reduce barriers to the use of eHealth applications, and ultimately strengthen the impact of hybrid prevention models.

However, further research is needed to fully understand the relationships between eHealth literacy, psychosocial characteristics, and the actual use of medical apps. Moreover, future studies should investigate the interplay between psychosocial characteristics and social determinants of health to inform integrated intervention strategies. To strengthen the interpretability and practical relevance of eHealth literacy measures, future research should also examine how self-reported eHealth literacy relates to actual digital competencies and behavioral outcomes and work toward the validation of clinically meaningful categories to support actionable use in health care settings. In this context, Ecological Momentary Assessment methods could be applied to capture real-time patterns of digital engagement in naturalistic settings, thereby complementing self-report data and enhancing ecological validity. Longitudinal designs will be essential to determine causal pathways and to assess how tailored interventions can improve both eHealth literacy and overall health outcomes. Moreover, expanding research to diverse populations will be essential to ensure that eHealth interventions are accessible to all. By continuing to refine our understanding of these dynamics, we can move toward more effective, inclusive, and person-centered prevention strategies.

## Supplementary material

10.2196/73697 Multimedia Appendix 1 Background information on the study.

10.2196/73697 Multimedia Appendix 2 Overview of instruments used for data collection, including the full item set of the eHLUS.

10.2196/73697 Multimedia Appendix 3 Statistical analysis.

10.2196/73697 Checklist 1 Checklist for reporting results of Internet e-surveys (CHERRIES).

## Abbreviations

DASS-21: Depression Anxiety Stress Scale with 21 Items
Dvs: dependent variables
eHEALS: eHealth Literacy Scale
eHLUS: eHealth Literacy and Use Scale
GLM: general linear model
KMO: Kaiser-Meyer-Olkin measure of sampling adequacy
MANOVA: multivariate analysis of variance
PCA: principal component analysis
QoL: Quality of Life
SSS: Subjective Socioeconomic Status (MacArthur Scale)
SWOP-K9: Self-Efficacy, Optimism, Pessimism Scale with 9 Items
TmeHL: Transactional Model of eHealth Literacy
WAI: Work Ability Index
WCSS: within-cluster sum of squares
