# Supplementary material for: Association Between Psychosocial Characteristics and eHealth Literacy: Cross-Sectional Study of Hybrid Secondary Prevention in Mental Health
Source: JMIR Ment Health. 2025 Oct 20;12:e73697. doi: 10.2196/73697 (PMC12536944; doi:10.2196/73697)
Supplement: Multimedia Appendix 1 [file mental-v12-e73697-s001.docx]

Table S1: Study Background

| **Aspect** | **Details** |
| --- | --- |
| **Project Name** | Development, Piloting, and Evaluation of an App-Supported Psychosocial Prevention Intervention (German acronym: PE³PP). |
| **Funding** | German Federal Ministry of Labor and Social Affairs |
| **Duration** | October 2021 - October 2026 |
| **Objective** | Promoting and strengthening participation in working life through a secondary app-based prevention intervention being developed, piloted and evaluated called 'RV Fit Mental Health'. |
| **Target Group** | The intervention explicitly focused on mental health problems related to or exacerbated by the work environment, such as affective disorders, phobic and other anxiety disorders, adjustment disorders, somatoform disorders and burn-out. |
| **Research Design** | Three-stage parallel mixed-methods design investigating the development (Stage 1), piloting (Stage 2), and evaluation (Stage 3) of the ‘RV Fit Mental Health’ intervention. |
| **Intervention Components** | A two-week initial inpatient phase and a twelve-week training phase supported by the 'Minddistrict' app and therapeutic support provided via the app. |
| **App Functionalities** | Provides various content and modules of the intervention, including exercises, instructions, training, and seminar content in text, video, and audio formats; ensures a digital connection between participants and clinic staff |
| **App Classification** | Minddistrict app is classified as a ‘medical app’ for the intervention RV Fit Mental Health |
| **Study Protocol Publication** | Published under: Stephan J, Gehrmann J, Stullich A for the PE³PP study group, et al. Development, piloting and evaluation of an app-supported psychosocial prevention intervention to strengthen participation in working life: a study protocol of a mixed-methods approach. BMJ Open 2024;14:e081390. doi: 10.1136/bmjopen-2023-081390 |
| **Research Team** | Technical University of Munich, TUM School of Medicine and Health, Chair of Social Determinants of Health |
| **Intervention Participants** | Expected 960 participants from September 2023 to September 2026 |
| **Collaborating Partners** | German Pension Insurance Central Germany, AOK PLUS, Median Klinik Bad Gottleuba, SRH Medinet Burgenlandklinik |
| **Ethical Approval** | Ethical approval obtained from the Ethics Committee at the TUM School of Medicine and Health (2022-523 S-SR; 2023-316 S-SB). |
| **Study Registration** | Trial registration numbers DRKS00030818 and DRKS00033080 |
| Note. Adapted from: Stephan J, Gehrmann J, Dehner JC, Stullich A, Richter M. (2025). Development and validation of the eHealth Literacy and Use Scale (eHLUS) to measure medical app literacy. Public Health, 240, 27–32. https://doi.org/10.1016/j.puhe.2024.12.057. Licensed under CC BY 4.0 (https://creativecommons.org/licenses/by/4.0/). | |
