# Supplementary material for: Association Between Psychosocial Characteristics and eHealth Literacy: Cross-Sectional Study of Hybrid Secondary Prevention in Mental Health
Source: JMIR Ment Health. 2025 Oct 20;12:e73697. doi: 10.2196/73697 (PMC12536944; doi:10.2196/73697)
Supplement: Multimedia Appendix 2 [file mental-v12-e73697-s002.docx]

Table S1: Overview of the instruments used to assess psychosocial characteristics and eHealth literacy.

| Instrument full name | Measurement | Number of items | Instrument abbreviation | Validity and reliability | Source | |
| --- | --- | --- | --- | --- | --- | --- |
| Depression Anxiety Stress Scale-21 | depression, anxiety, stress | 21 | DASS-21 | The participants in the total sample (n=307) can be divided into several groups based on their diagnosis and a group of people from the general population.  Panic disorder with or without agoraphobia: n=67, obsessive-compulsive disorder: n=54, social phobia: n=74, specific phobia: n=17, major depressive disorder: n=46, and non-clinical volunteers: n=49.  Internal consistency values for each subscale: depression α=0.94, anxiety α=0.87, and stress α=0.91. | [1] | |
|  |  |  | DASS-21 (German Version) | German Version of the DASS-21. Participants of the total sample size (n=714) was divided into two groups:  Pain patients: n=301, adults in the general population: n=413.  Internal consistency values for each subscale: depression α=0.88, anxiety α=0.76-0.80, stress α=0.86-0.87. | [2] | |
| eHealth Literacy Scale | ability to find, evaluate, and apply health information from the internet | 8 | eHEALS | The total sample size comprised 664 participants aged 13–21 years.  The internal consistency of the overall scale is α=0.88. | [3] | |
|  |  |  | eHEALS | The total sample (n=866) consisted of people aged 50 years and over who were recruited for the Bone Power Study via two online portals.  The internal consistency of the overall scale is α=0.94. | [4] | |
|  |  |  | G-eHEALS (German Version) | This is a version of the eHEALS that has been translated into German. The sample (n=327) consisted of German students.  Reliability was tested using a 2-factor model, which resulted in α=0.88 for the information search dimension and α=0.82 for the information evaluation dimension. | [5] | |
| eHealth Literacy and Use Scale | eHealth engagement, autnomouse use and technical access, eHealth literacy | 14 | eHLUS | The total sample (n = 127) comprised German employees between the ages of 27 and 64 with mental health conditions who took part in a secondary prevention program targeting mental health.  The internal consistency of the overall scale is α=0.91. The three dimensions showed good to excellent internal consistency: eHealth engagement (α = 0.925), autonomous use and technical access (α = 0.855), and eHealth literacy (α = 0.839) | [6] | |
| Self-efficacy-optimism-pessimism questionnaire | self-efficacy, optimism, pessimism | 9 | SWOP-K9 | The SWOP-K9 consists of two questionnaires that were combined and shortened [7]. These are the self-efficacy questionnaire [8] and the optimism questionnaire [9].  The resulting SWOP-K9 was compared with the original questionnaires in five different clinical samples with n=726 inpatients. Statistical validation revealed good to optimal scores [7].  The reliability of the SWOP-K9 is based on the testing of the individual questionnaires on self-efficacy with an internal consistency of α=0.86 (n=208) and on optimism with an internal consistency of α=0.76 (n=624). According to the creators, the questionnaire is valid for adults. | [7–9] | |
| WHO Quality of Life Instrument, Short Form | physical health, psychological health, social relationships, environment | 26 | WHOQOL-BREF | The total sample (n=11830) included individuals from 23 countries. Of these, n=2308 people from Germany participated. Participants were adults recruited from a variety of inpatient and outpatient healthcare facilities as well as from the general population.  For the German-speaking sample, the internal consistency for the subdomains was as follows: physical health α=0.88, mental health α=0.83, social relationships α=0.76, and environment α=0.78. | [10] | |
| Work Ability Index | Ability to work, physical and mental health, job require-ment/ satisfaction | 7 | WAI | The sample size (n=3968) comprises people employed in Germany.  The internal consistency of the scale was α= 0.75. | [11] | |
| *α = Cronbach's alpha*  Note. Adapted from: Stephan J, Gehrmann J, Stullich A, Hoffmann L, Richter M. (2024). Development, piloting and evaluation of an app-supported psychosocial prevention intervention to strengthen participation in working life: a study protocol of a mixed-methods approach. BMJ Open, 14(2), e081390. https://doi.org/10.1136/bmjopen-2023-081390. Licensed under CC BY 4.0 (https://creativecommons.org/licenses/by/4.0/). One column (eHealth Literacy and Use Scale) was added by the authors of the present work. | | | | | |  |

Table S2: The 14 items of the eHLUS with validated German wording, author-translated English version, and assigned dimensions.

| Item No. | Item in German | Item in English | Dimension | |
| --- | --- | --- | --- | --- |
| 1 | Digitale Gesundheitsanwendungen können im Allgemeinen dazu beitragen meine Gesundheit zu fördern. | Digital health applications can generally help improve my health. | eHealth Engagement | |
| 2 | Ich finde es attraktiv, dass ich digitale Gesundheitsanwendungen selbstständig anwenden kann. | I find it appealing that I can use digital health applications independently. | eHealth Engagement | |
| 3 | Ich bin motiviert digitale Gesundheitsanwendungen im Alltag zu nutzen. | I am motivated to use digital health applications in everyday life. | eHealth Engagement | |
| 4 | Eine Nutzung digitaler Gesundheitsanwendungen ist für mich einfach in den Alltag zu integrieren. | Using digital health applications is easy to integrate into my daily life. | eHealth Engagement | |
| 5 | Ich habe immer Zugriff auf die notwendige Hardware (z. B. Smartphone, PC), um digitale Gesundheitsanwendungen zu nutzen. | I always have access to the necessary hardware (e.g., smartphone, PC) to use digital health applications. | Autonomous Use and Technical Access | |
| 6 | Ich habe immer Zugang zu stabilem und zuverlässigem Internet. | I always have access to stable and reliable internet. | Autonomous Use and Technical Access | |
| 7 | Ich weiß, wie ich das Internet nutzen kann, um Antworten auf meine Fragen rund um das Thema Gesundheit zu bekommen. | I know how to use the internet to get answers to my health-related questions. | eHealth Literacy | |
| 8 | Ich weiß, wo ich im Internet nützliche Gesundheitsinformationen finden kann (z. B. Bundeszentrale für gesundheitliche Aufklärung, Bundesministerium für Gesundheit). | I know where to find useful health information on the internet (e.g., Federal Center for Health Education, Federal Ministry of Health). | eHealth Literacy | |
| 9 | Ich bin in der Lage Informationen, die meine Gesundheit betreffen, kritisch zu bewerten. | I am able to critically evaluate information related to my health. | eHealth Literacy | |
| 10 | Ich kann im Internet zuverlässige von fragwürdigen Informationen und Quellen, die meine Gesundheit betreffen, unterscheiden. | I can distinguish reliable from questionable information and sources regarding my health on the internet. | eHealth Literacy | |
| 11 | Ich fühle mich in der Anwendung digitaler Geräte (Smartphone, PC usw.) sicher. | I feel confident using digital devices (smartphone, PC, etc.). | Autonomous Use and Technical Access | |
| 12 | Ich bin in der Lage, digitale Gesundheitsanwendungen ohne Hilfe von Dritten anzuwenden. | I am able to use digital health applications without help from others. | Autonomous Use and Technical Access | |
| 13 | Wenn ich Hilfe bei der Nutzung von Apps oder digitaler Technik benötige, erhalte ich jederzeit Unterstützung aus meinem sozialen Umfeld. | If I need help using apps or digital technology, I can always get support from my social environment. | Autonomous Use and Technical Access | |
| 14 | Ich denke, dass meine Daten bei digitalen Gesundheitsanwendungen ausreichend geschützt sind. | I think my data is sufficiently protected when using digital health applications. | Autonomous Use and Technical Access | |
| Note. Adapted from: Stephan J, Gehrmann J, Dehner JC, Stullich A, Richter M. (2025). Development and validation of the eHealth Literacy and Use Scale (eHLUS) to measure medical app literacy. Public Health, 240, 27–32. https://doi.org/10.1016/j.puhe.2024.12.057. Licensed under CC BY 4.0 (https://creativecommons.org/licenses/by/4.0/). The columns “Factor” and “Factor loadings” were removed by the authors of the present work. | | | |  |

References

1. Antony MM, Bieling PJ, Cox BJ, Enns MW, Swinson RP. Psychometric properties of the 42-item and 21-item versions of the Depression Anxiety Stress Scales in clinical groups and a community sample. Psychological Assessment 1998;10(2):176-181. doi:10.1037/1040-3590.10.2.176

2. Nilges P, Essau C. DASS. Depressions-Angst-Stress-Skalen - deutschsprachige Kurzfassung; 2021.

3. Norman CD, Skinner HA. eHEALS: The eHealth Literacy Scale. J Med Internet Res 2006;8(4):e27. PMID:17213046

4. Chung S-Y, Nahm E-S. Testing reliability and validity of the eHealth Literacy Scale (eHEALS) for older adults recruited online. Comput Inform Nurs 2015;33(4):150-156. PMID:25783223

5. Soellner R, Huber S, Reder M. The Concept of eHealth Literacy and Its Measurement. Journal of Media Psychology 2014;26(1):29-38. doi:10.1027/1864-1105/a000104

6. Stephan J, Gehrmann J, Dehner JC, Stullich A, Richter M. Development and validation of the eHealth Literacy and Use Scale (eHLUS) to measure medical app literacy. Public Health 2025;240:27-32. doi:10.1016/j.puhe.2024.12.057

7. Scholler G, Fliege H, Klapp BF. SWOP-K9 - Fragebogen zu Selbstwirksamkeit-Optimismus-Pessimismus Kurzform 1999 URL: https://doi.org/10.23668/psycharchives.6595 [accessed 2025-08-04].

8. Schwarzer R, Jerusalem M, Weinman J, Wright S, Johnston M. Generalized Self-Efficacy Scale. Measures in Health Psychology: A User's Portfolio. Causal and control beliefs Windsor 1995.

9. Scheier MF, Carver CS. Optimism, coping, and health: assessment and implications of generalized outcome expectancies. Health Psychol 1985;4(3):219-247. PMID:4029106

10. Skevington SM, Lotfy M, O'Connell KA. The World Health Organization's WHOQOL-BREF quality of life assessment: psychometric properties and results of the international field trial. A report from the WHOQOL group. Qual Life Res 2004;13(2):299-310. PMID:15085902

11. Freyer M. Eine Konstruktvalidierung des Work Ability Index anhand einer repräsentativen Stichprobe von Erwerbstätigen in Deutschland 2019 URL: https://www.baua.de/DE/Angebote/Publikationen/Berichte/F2250-3 [accessed 2025-08-04].
