# Supplementary material for: Association Between Psychosocial Characteristics and eHealth Literacy: Cross-Sectional Study of Hybrid Secondary Prevention in Mental Health
Source: JMIR Ment Health. 2025 Oct 20;12:e73697. doi: 10.2196/73697 (PMC12536944; doi:10.2196/73697)
Supplement: Multimedia Appendix 3 [file mental-v12-e73697-s003.docx]

Table S1: Summary of between-subjects effects (GLM results).

| **Dependent variable** | **Source** | **F value** | ***P*** |
| --- | --- | --- | --- |
| eHEALS total score | Age | 18.812 | <.001 |
|  | SSS | 4.848 | .03 |
|  | Cluster | 2.346 | .08 |
| eHLUS total score | Age | 24.515 | <.001 |
|  | SSS | 8.560 | .004 |
|  | Cluster | 3.909 | .01 |
| Dimension: eHealth engagement | Age | 6.419 | .01 |
|  | SSS | 2.052 | .15 |
|  | Cluster | 1.704 | .17 |
| Dimension: autonomous use and technical access | Age | 33.152 | <.001 |
|  | SSS | 6.422 | .01 |
|  | Cluster | 4.658 | .004 |
| Dimension: eHealth literacy | Age | 14.753 | <.001 |
|  | SSS | 10.757 | .001 |
|  | Cluster | 1.638 | .18 |
| *P*=probability value; SSS=Subjective Socioeconomic Status | | | |

Table S2: Multivariate test statistics for associations of cluster membership, SSS, and age with eHealth literacy dimensions (GLM results).

| **Effect** | **Wilks' lambda** | **Pillai's Trace** | **Hotelling's Trace** | **Roy's Largest Root** | **F value** | ***P* value** | |
| --- | --- | --- | --- | --- | --- | --- | --- |
| Age | 0.826 | 0.174 | 0.210 | 0.210 | 6.844 | <.001 | |
| SSS | 0.931 | 0.069 | 0.074 | 0.074 | 2.416 | .04 | |
| Cluster | 0.898 | 0.104 | 0.112 | 0.088 | 1.185 | .28 | |
| F value=F-statistic (a test statistic used in hypothesis testing to evaluate group differences); *P*=probability value; SSS=Subjective Socioeconomic Status | | | | | | |  |
